# Development and validation of a nomogram for predicting 28-day mortality in patients with ischemic stroke

**DOI:** 10.1371/journal.pone.0302227

**Published:** 2024-04-24

**Authors:** Lingyan Fang, Menglu Zhou, Fengkai Mao, Mengyuan Diao, Wei Hu, Guangyong Jin

**Affiliations:** 1 Department of Critical Care Medicine, Hangzhou First People’s Hospital, Hangzhou, Zhejiang, China; 2 Department of Neurology, Affiliated Hospital of Hangzhou Normal University, Hangzhou, Zhejiang, China; 3 Affiliated Hospital of Hangzhou Normal University, Hangzhou Normal University, Hangzhou, Zhejiang, China; Union Hospital, Tongji Medical College, Huazhong University of Science and Technology, CHINA

## Abstract

**Background/aim:**

We aimed to construct a validated nomogram model for predicting short-term (28-day) ischemic stroke mortality among critically ill populations.

**Materials and methods:**

We collected raw data from the Medical Information Mart for Intensive Care IV database, a comprehensive repository renowned for its depth and breadth in critical care information. Subsequently, a rigorous analytical framework was employed, incorporating a 10-fold cross-validation procedure to ensure robustness and reliability. Leveraging advanced statistical methodologies, specifically the least absolute shrinkage and selection operator regression, variables pertinent to 28-day mortality in ischemic stroke were meticulously screened. Next, binary logistic regression was utilized to establish nomogram, then applied concordance index to evaluate discrimination of the prediction models. Predictive performance of the nomogram was assessed by integrated discrimination improvement (IDI) and net reclassification index (NRI). Additionally, we generated calibration curves to assess calibrating ability. Finally, we evaluated the nomogram’s net clinical benefit using decision curve analysis (DCA), in comparison with scoring systems clinically applied under common conditions.

**Results:**

A total of 2089 individuals were identified and assigned into training (n = 1443) or validation (n = 646) cohorts. Various identified risk factors, including age, ethnicity, marital status, underlying metastatic solid tumor, Charlson comorbidity index, heart rate, Glasgow coma scale, glucose concentrations, white blood cells, sodium concentrations, potassium concentrations, mechanical ventilation, use of heparin and mannitol, were associated with short-term (28-day) mortality in ischemic stroke individuals. A concordance index of 0.834 was obtained in the training dataset, indicating that our nomogram had good discriminating ability. Results of IDI and NRI in both cohorts proved that our nomogram had positive improvement of predictive performance, compared to other scoring systems. The actual and predicted incidence of mortality showed favorable concordance on calibration curves (P > 0.05). DCA curves revealed that, compared with scoring systems clinically used under common conditions, the constructed nomogram yielded a greater net clinical benefit.

**Conclusions:**

Utilizing a comprehensive array of fourteen readily accessible variables, a prognostic nomogram was meticulously formulated and rigorously validated to provide precise prognostication of short-term mortality within the ischemic stroke cohort.

## Introduction

Stroke represents a significant healthcare challenge on a global scale. Despite the stable morbidity and declining mortality, stroke, stroke survivors, and deaths due to stroke have increased stably over the past two decades [1]. Ischemic stroke, a destructive disease and a common cause of stroke, is definitely a dominating and remarkable cause of disability and death, subsequently bringing huge burden to patients, families, society and countries [2–4]. Researchers have estimated that approximately $1841 billion will be spent on stroke-related medical expenses between 2012 and 2030 [5]. Therefore, it is important to identify prognostic factors and distinguish ischemic stroke patients with adverse outcome. Studies have identified several factors associated with death of ischemic stroke patients, including age [6], cancer [7], marital status, underlying metastatic solid tumor, heart rate, Glasgow Coma Scale (GCS) [8], glucose, white blood cells (WBC) [9], mechanical ventilation (MV), and osmotic therapy [10].

Emerging as formidable instruments for prognostication in the context of ischemic stroke, nomogram models wield significant potency in predicting survival outcomes. For example, researchers developed a dynamic nomogram, based on National Institute of Health Stroke Scale (NIHSS), to predict adverse outcomes [11]. Such models have enabled comprehensive evaluation of mortality, facilitated communication of medical conditions, and subsequently mediated effective prevention of medical disputes [12]. Few studies have reported development of nomogram models for prediction of ischemic stroke mortality. For instance, utilizing the ratio of triglycerides to high-density lipoprotein cholesterol, a prognostic nomogram was developed to ascertain mortality within a three-month timeframe. Regrettably, the model exhibited a suboptimal concordance index (C-index) of 0.684, indicative of its limited predictive accuracy [13]. In light of the advancing global demographic shift towards an aging population, our preceding investigation culminated in the formulation of a meticulously designed nomogram [14]. This nomogram exhibits a remarkable capacity to precisely prognosticate the near-term mortality likelihood among elderly cohorts afflicted by ischemic stroke [14]. Interestingly, a nomogram has been successfully developed using point-of-care information to predict 10-year stroke mortality [7]. Another model was developed using 767 stroke patients and found to effectively predict stroke mortality at 1, 6, and 12 months after stroke [15].

With the objective of synthesizing disparate risk factors into a comprehensive predictive model, our study aimed to construct a nomogram tailored to forecast 28-day mortality in ischemic stroke. Leveraging readily available clinical data from the Medical Information Mart for Intensive Care IV (MIMIC-IV) database, our endeavor sought to enhance prognostic precision in this critical clinical domain.

## Materials and methods

### Data source & ethical statement

All raw data were derived from MIMIC-IV (version 2.1), which comprised cataloging of more than 70000 individuals in Beth Israel Deaconess Medical Center (BIDMC, Boston, MA), while those patients were hospitalized in the intensive care unit (ICU) from 2008 to 2019. We accessed the database for research purposes on December 28, 2022. The obtained information included recorded signs, laboratory indicators, and medications information, among others [8]. Institutional review boards at the Massachusetts Institute of Technology (MIT, Cambridge, MA) and BIDMC provided approval for the establishment of the MIMIC-IV database. The project’s lead author, designated as GJ, possesses credentials as a verified user on PhysioNet, an esteemed platform dedicated to the sharing and analysis of physiological data. Furthermore, author GJ has successfully completed a rigorous training course in human subject research, as evidenced by certification number 46141344. Then, MIMIC-IV was installed in a local PostgreSQL database on windows, and data extracted using PostgreSQL tools (version 14.2.1, The PostgreSQL Global Development Group, California, USA).

In accordance with prevailing national regulations and institutional guidelines, the necessity for informed consent was duly waived, given the retrospective design inherent to this investigation. Approval for the conduct of this study was meticulously sought and obtained from the Institutional Review Board of Hangzhou First People’s Hospital, following a thorough review process ensuring adherence to ethical standards and regulatory mandates (approval number: ZN-20231120-0265-01). The authors were unable to obtain information that could identify individual participants during or after data collection.

### Study subjects and data extraction

We recruited ischemic stroke individuals with first admission to the ICU, as described in S1 Fig and our previous study [8]. Data from 2nd and later ICU stays were excluded. Ischemic stroke was identified based on the guidelines of the International Classification of Disease (ICD) (both version 9 and version 10). We also excluded duplicate records during first ICU stay, as well as those from minor patients (<18 years old) and elderly patients (>89 years old). In order to address potential sources of heterogeneity within our study population, individuals with an ICU length of stay (LOS) of less than one day were deliberately excluded. Subsequently, a cohort comprising 2089 patients diagnosed with ischemic stroke was meticulously assembled. Employing a methodological approach consistent with our prior investigation [8], this cohort was randomly partitioned into distinct developing and validation sets at a ratio of 7:3.

In our study, pertinent variables were meticulously extracted from the dataset, encompassing a comprehensive array of demographic, clinical, and therapeutic factors. Specifically, variables including age, gender, weight, marital status, ethnicity, type of admission, first care unit, as well as key temporal parameters such as hospital and ICU admission/discharge times, and in the unfortunate event, time of demise were meticulously documented. Leveraging the robust infrastructure of the PostgreSQL platform, MIMIC-IV concepts—valuable abstractions derived from the raw dataset—were meticulously generated, ensuring a standardized and structured representation of critical care data. Furthermore, a multitude of established clinical scoring systems including the Acute Physiology Score III (APS III), Simplified Acute Physiology Score II (SAPS II), Sequential Organ Failure Assessment (SOFA), Oxford Acute Severity of Illness Score (OASIS), Logistic Organ Dysfunction System (LODS), Glasgow Coma Scale (GCS), and a comprehensive array of vital signs, biochemical indicators, and hematologic parameters obtained on the first day of ICU admission were meticulously curated. Additionally, comorbidities and the Charlson Comorbidity Index (CCI) were meticulously identified utilizing the Charlson table within the MIMIC-IV dataset. To capture therapeutic interventions, meticulous documentation was undertaken to ascertain whether patients received mechanical ventilation, underwent endovascular obstruction removal, or were administered various pharmacologic agents such as antiplatelets, heparin, alteplase, albumin, furosemide, mannitol, and vasopressors within the initial 24 hours of ICU admission. To ensure granularity and accuracy, data points obtained multiple times during this critical period were systematically extracted using MIMIC concepts. For each variable of interest, statistical descriptors including minimum, maximum, and when available, average values were meticulously computed, facilitating subsequent comprehensive analyses.

### Statistical analysis

The extracted data were saved in csv format on a local computer, and the variables pre-processed using Stata software. Outliers were detected using histogram and winsorized using winsor2 command in Stata software (replace cuts 0.5% and 99.5%). Multiple imputations were conducted to fill in the missing values. R software was used to randomly assign individuals to either the developing or validation datasets. The following statistical methods were applied to assess differences between the training and validation datasets: (1) Skewness and kurtosis tests were used to test normality continuous variables. (2) We applied Mann-Whitney U-test to assess differences between the training and validation cohorts if continuous variables were not normally distributed, and the results were described as median and interquartile range (IQR); (3) A number with percentage was applied to present categorical variables, which was tested by Chi-square test.

The statistical software R was employed for subsequent analyses. Utilizing a 10-fold cross-validation procedure in conjunction with the least absolute shrinkage and selection operator (LASSO) regression, latent variables were discerned from the extracted data within the training set, consistent with prior methodology [8]. Binary logistic regression, with the 28-day survival state as the dependent variable, was subsequently conducted. Additionally, a nomogram was constructed to forecast the 28-day mortality of ischemic stroke patients subsequent to their admission to the ICU. Comparative analysis of the nomogram’s predictive performance was undertaken against alternative logistic regression models based on prognostic scores. Discriminatory capacity of these models was assessed using the C-index across both datasets. Models yielding a C-index exceeding 0.7 were deemed to exhibit favorable discriminatory ability [16]. Like the other discrimination metrics, improvement of predictive performance on different models were assessed by integrated discrimination improvement (IDI), as well as index of net reclassification (NRI) [17]. Next, we employed ‘val.prob’ function to generate calibration curves for evaluation of model accuracy on both data sets. In order to assess clinical benefits and usefulness, we completed decision curve analysis (DCA) targeting the training set. Statistical procedures were performed in Stata software (version 17.0, Stata Corporation LLC, College Station, USA) (https://www.stata.com) as well as in R software (version 4.2.1, R Foundation for Statistical Computing, Vienna, Austria), using packages including rms, car, glmnet, pROC, regplot, PredictABEL and rmda data packages. In this study, a p-value <0.05 was used as the criterion for statistical significance.

## Results

### Baseline characteristics

2089 ischemic stroke individuals were identified, and randomly assigned into either the training (1443) or validation (646) cohorts. Profiles of patient characteristics between the cohorts are summarized in S1 Table and our previous study [8]. Our meticulous examination revealed no statistically discernible disparities among the characteristics of patients comprising the training and validation cohorts (P > 0.05), thereby indicating a judicious grouping of all individuals afflicted with ischemic stroke.

### Variable selection and nomogram construction

**Fig 1:**
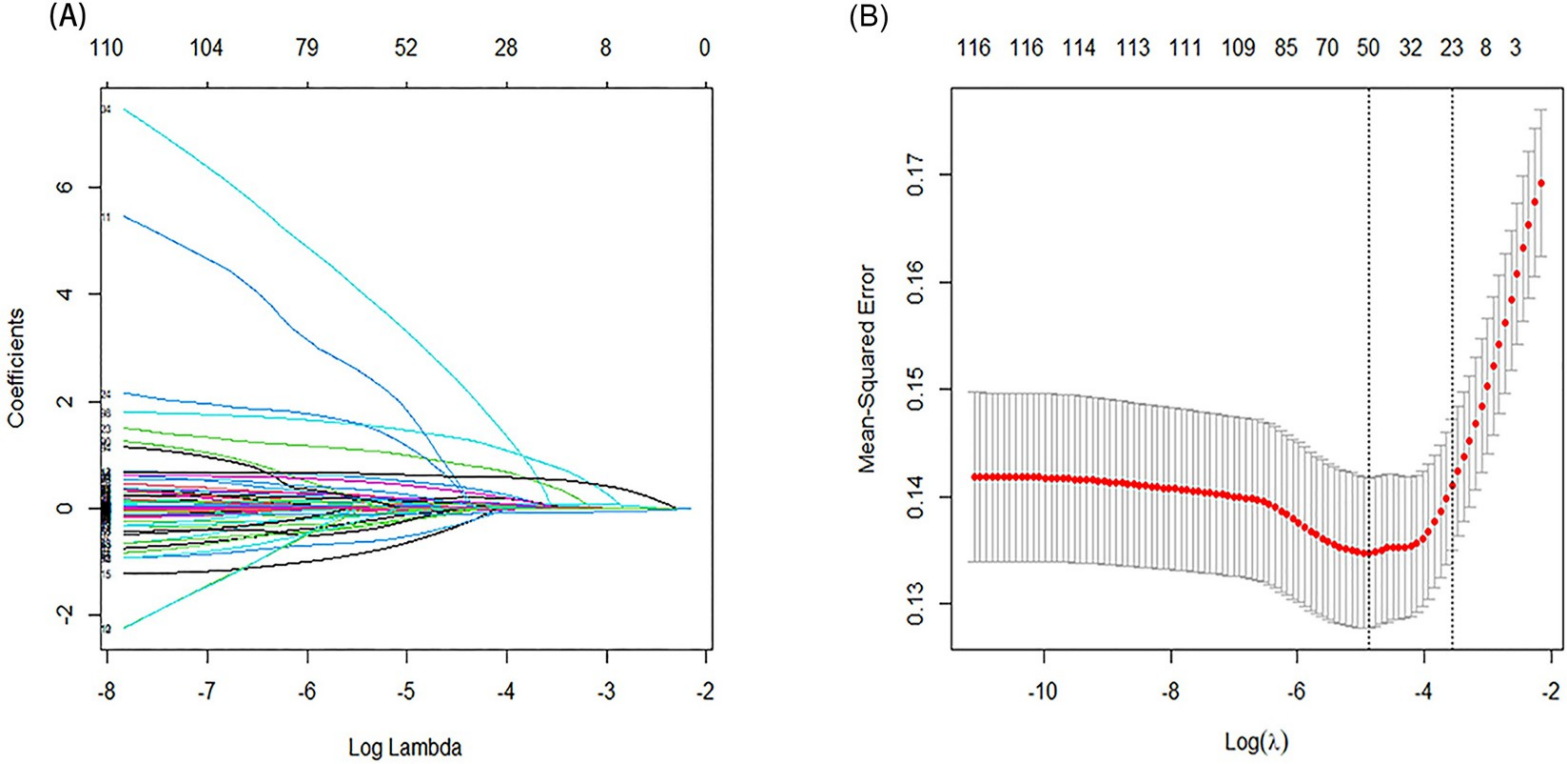
Selection of clinical features by least absolute shrinkage and selection operator (LASSO) regression and 10-fold cross-validation. (A) Visual plot of the relationship between coefficients for 117 features and the lambda. As lambda increased, the coefficient of each feature gradually tended to zero; (B) Curve of 10-fold cross-validation in the LASSO regression. The dotted vertical line on the left reflected the number of features and optimal log (lambda) corresponding to the smallest mean squared error (λ = 0.007716015). With one standard error criteria of optimal log (lambda), the dotted vertical line on the right reflected the model constructed with 23 variables was relatively accurate and simple (λ = 0.02838243). λ, lambda.

**Fig 2:**
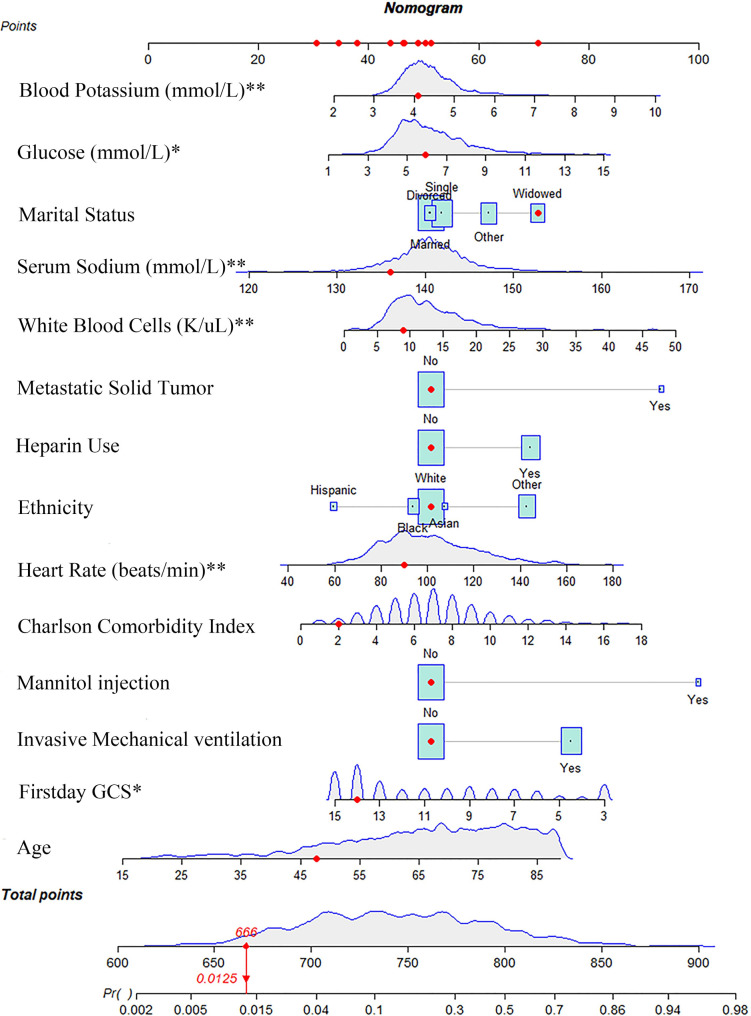
Nomogram for predicting 28-day mortality in patients with ischemic stroke. The red dot represented the example of a patient. We present the case of a 48-year-old widowed patient of white ethnicity, with no prior history of metabolic solid tumors, who was admitted to the intensive care unit (ICU) with a Charlson comorbidity index of 2. The patient did not receive invasive mechanical ventilation, heparin, or mannitol on the first day of admission. Upon initial evaluation, the patient’s Glasgow Coma Scale (GCS) score was 14 minutes, and the fastest heart rate recorded was 90 beats/min. The patient’s minimum blood glucose level was 5.94mmol/L, while the highest white blood cell count was 9K/ul. The patient’s highest recorded blood potassium level was 4.1mmol/L, while the highest recorded blood sodium level was 136mmol/L. The sum (666) of these points was located on the total points line, and a solid red line was drawn down to the survival axis to determine the risk probability of 28-day mortality (1.25%). *: the min value of indicators on the firstday of ICU stay; **: the max value of indicators on the firstday of ICU stay.

Selection of clinical features were shown in Fig 1A and 1B using LASSO regression. A total of 23 underlying variables, namely age, ethnicity type (Hispanics, black, Asian, other), marital status (single, widowed, divorced, other), type of first care unit, malignant cancer (yes), metastatic solid tumor (yes), CCI, GCS, heart rate, respiratory rate, temperature, glucose, white blood cells (WBC), blood urea nitrogen (BUN), anion gap (AG), sodium concentrations, potassium concentrations, use of MV (yes), phenylephrine injection (yes), vasopressin injection (yes), heparin use (yes), dipyridamole use (yes) and mannitol injection (yes), were selected for multivariate analysis. Subsequently, fourteen factors were found to be associated with 28-day mortality after first admission to the ICU (P < 0.05), as shown in Table 1, and those fourteen variables were used to construct the nomogram (Fig 2).

**Table 1:** The multivariable logistic regression analyses of independent risk factors for 28-day mortality in patients with ischemic stroke in the training cohort.

Variables	OR	95% CI	P-value
Age (years)	1.036	1.021–1.051	<0.001
Ethnicity (Hispanic)	0.562	0.192–1.430	0.255
Ethnicity (Black)	0.903	0.529–1.503	0.701
Ethnicity (Asian)	1.088	0.365–2.901	0.873
Ethnicity (Other)	1.774	1.154–2.712	0.008
Marital status (Single)	1.067	0.702–1.611	0.761
Marital status (Widowed)	1.888	1.181–3.008	0.008
Marital status (Divorced)	0.995	0.517–1.844	0.988
Marital status (Other)	1.412	0.856–2.334	0.176
Metastatic solid tumor (Yes)	3.931	1.833–8.475	<0.001
Charlson comorbidity index	1.119	1.038–1.207	0.003
Heart rate (beats/min) **	1.014	1.007–1.021	<0.001
Glucose (mmol/L) *	1.124	1.039–1.216	0.003
White Blood Cells (K/μL) **	1.040	1.017–1.063	<0.001
Serum sodium (mmol/L) **	1.054	1.021–1.088	0.001
Blood potassium (mmol/L) **	1.270	1.061–1.516	0.008
Mannitol use (Yes)	4.904	2.557–9.485	<0.001
Heparin use (Yes)	1.808	1.313–2.488	<0.001
Mechanical ventilation (Yes)	2.306	1.672–3.187	<0.001
Firstday GCS*	0.875	0.841–0.910	<0.001

OR, odd ratio; CI, confidence interval; GCS, Glasgow Coma Scale

*: the min value of indicators on the firstday of ICU stay

**: the max value of indicators on the firstday of ICU stay.

### Nomogram evaluation and validation

**Fig 3:**
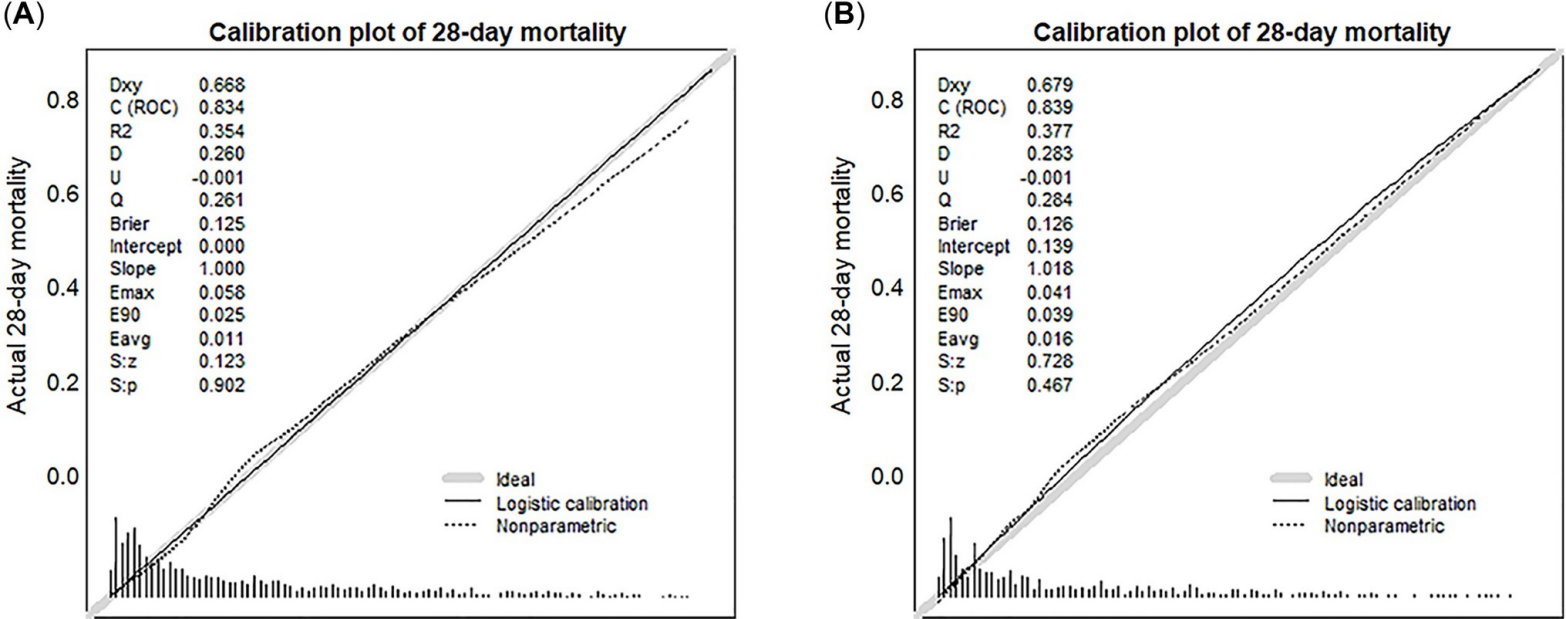
Calibration curve of constructed nomogram in the training set (A) and validation set (B). The Receiver Operating Characteristic (ROC) curve analysis of the training set yielded an area under the curve (AUC) of 0.834, while the validation set exhibited an AUC of 0.839. The predicted and actual 28-day mortality was no statistical significance in both training and validation set (All P > 0.05; P = 0.902 in the training set and P = 0.467 in the validation set).

Our nomogram’ C-index in development dataset was 0.834 (95% CI: 0.810, 0.859), which demonstrated good accuracy of the constructed nomogram for predicting prognosis in ischemic stroke. A comparable C-index (0.839, 95% CI: 0.804, 0.874) was obtained in validation set. Our nomogram displayed a higher C-index in both data sets than those reported in scoring systems clinically applied under common conditions (Table 2), suggesting it had better performance in predicting outcomes than these scoring systems clinically applied under common conditions. Similarly, results of IDI and NRI in both cohorts proved that our nomogram had positive improvement of predictive performance, compared to scoring systems clinically applied under common conditions (Table 3). Collectively, these results indicated that our nomogram was superior at predicting the probability of 28-day mortality than commonly used systems. Model calibration results revealed that our nomogram accurately predicted probabilities in each cohort, relative to actual outcomes (Fig 3A and 3B; All P > 0.05).

**Table 2:** C-index of nomogram and critical care scoring systems in 28-day mortality prediction in ischemic stroke patients.

Models	Training cohort		Validation cohort	
	C-index	95% CI	C-index	95% CI
Nomogram	0.834	0.810‐0.859	0.839	0.804‐0.874
GCS	0.663	0.625‐0.701	0.710	0.655‐0.765
SOFA	0.687	0.656‐0.719	0.687	0.640‐0.733
APSIII	0.737	0.707‐0.768	0.763	0.719‐0.807
LODS	0.733	0.703‐0.763	0.745	0.700‐0.790
SAPSII	0.751	0.722‐0.780	0.746	0.703‐0.789
OASIS	0.744	0.715‐0.773	0.776	0.736‐0.816

C-index, concordance index; CI, confidence interval; GCS, Glasgow coma score; SOFA, sequential organ failure assessment; APS III, acute physiology score III; LODS, logistic organ dysfunction system; SAPS II, simplified acute physiology score II; OASIS, oxford acute severity of illness score.

**Table 3:** Comparison of NRI and IDI among models predicting 28-day mortality.

Index	Training cohort			Validation cohort		
	Estimate	95% CI	P-value	Estimate	95% CI	P-value
NRI (vs. GCS)	0.134	0.034‐0.233	0.009	0.267	0.122‐0.411	<0.001
NRI (vs. SOFA)	0.376	0.293‐0.460	<0.001	0.281	0.147‐0.415	<0.001
NRI (vs. APSIII)	0.142	0.048‐0.236	0.003	0.115	0.034‐0.265	0.130
NRI (vs. LODS)	0.191	0.099‐0.284	<0.001	0.228	0.085‐0.371	0.002
NRI (vs. SAPSII)	0.228	0.140‐0.316	<0.001	0.181	0.038‐0.324	0.013
NRI (vs. OASIS)	0.162	0.073‐0.251	<0.001	0.209	0.070‐0.348	0.003
IDI (vs. GCS)	0.120	0.090‐0.150	<0.001	0.113	0.072‐0.154	<0.001
IDI (vs. SOFA)	0.158	0.131‐0.184	<0.001	0.112	0.073‐0.151	<0.001
IDI (vs. APSIII)	0.087	0.056‐0.119	<0.001	0.084	0.042‐0.127	0.001
IDI (vs. LODS)	0.093	0.063‐0.122	<0.001	0.093	0.053‐0.132	<0.001
IDI (vs. SAPSII)	0.095	0.068‐0.123	<0.001	0.086	0.048‐0.124	<0.001
IDI (vs. OASIS)	0.088	0.060‐0.116	<0.001	0.084	0.047‐0.121	<0.001

NRI, net reclassification index; IDI, integrated discrimination improvement; CI, confidence interval; GCS, Glasgow coma score; SOFA, sequential organ failure assessment; APS III, acute physiology score III; LODS, logistic organ dysfunction system; SAPS II, simplified acute physiology score II; OASIS, oxford acute severity of illness score.

### Clinical value of the nomogram

**Fig 4:**
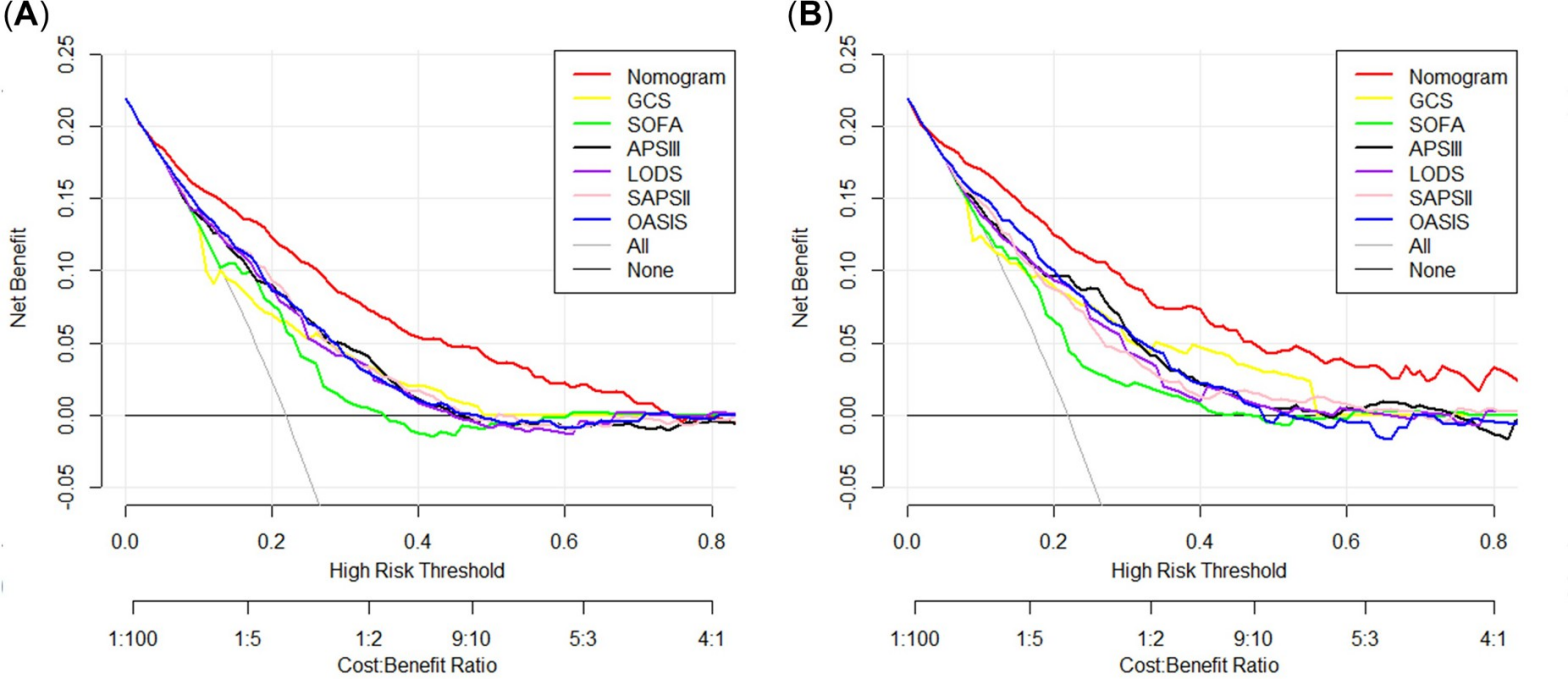
The decision curves analysis for constructed nomogram and models based on common clinical scoring systems in the training set (A) and validation set (B). The decision curves analysis demonstrates the performance of the constructed nomogram alongside models derived from conventional clinical scoring systems in both the training set (A) and validation set (B). The red line represents the outcomes of the constructed nomogram, indicating its superior performance. Notably, the utilization of the developed nomogram yields enhanced net benefits across a threshold probability range of 3% to 75%. GCS, Glasgow coma score; SOFA, sequential organ failure assessment; APS III, acute physiology score III; LODS, logistic organ dysfunction system; SAPS II, simplified acute physiology score II; OASIS, oxford acute severity of illness score.

Next, an analysis of the DCA curve was performed to assess the nomogram’s clinical value by determining severity scores in the training set. As a result, the constructed nomogram had greater net clinical benefits than scoring systems clinically applied under common conditions. Our nomogram’s clinical benefit was represented by the red line in Fig 4. Overall, our nomogram exhibited the best performance, while that based on SOFA score performed poorly.

The clinical benefit of our nomogram is vividly portrayed through the discernible trajectory of the red line depicted in Fig 4. In a comprehensive assessment of predictive efficacy, our nomogram emerged as the frontrunner, showcasing superlative performance metrics. Conversely, the model predicated on SOFA score demonstrated notably inferior performance characteristics (Fig 4), underscoring its limited predictive utility within the scope of our investigation.

## Discussion

In this study, 23 latent variables were successfully screened out using LASSO regression and 10-fold cross-validation. Fourteen of them were confirmed by multivariate logistic regression, associated with 28-day mortality among ischemic stroke individuals, and used to develop a nomogram for mortality prediction, namely age, ethnicity type, marital status, underlying metastatic solid tumor, CCI, heart rate, GCS, WBC, glucose concentrations, sodium concentrations, potassium concentrations, MV, use of heparin and mannitol injection. Analysis of the resultant C-index, NRI, IDI, calibration curves and DCA, revealed that our nomogram performed satisfactorily in both data sets. Notably, our nomogram was superior to those based on scoring systems clinically applied under common conditions with regards to discrimination net clinical benefits. Overall, these results indicate that the constructed nomogram has potential for application in clinical settings.

The use of clinical prediction models in stroke population has attracted considerable scholarly interest. For instance, a novel prognostic model was developed and aimed to predict late seizures after stroke [18]. Other scholars combined multiple models to construct a model for predicting stroke probability, which found to be 98.53% accurate [19]. Katharina et al. [20] developed and validated a model for identifying individuals who were at high-stake of ischemic stroke in the 12 months following noncardiac surgery. In addition, a risk score, established based on multi-center data, was found to efficiently predict long-term risk of ischemic stroke in antithrombotic individuals [21].

Several critical scoring systems have been previously applied to elucidate prognosis or severity, and achieved satisfactory results [22–25]. To date, however, whether they are applicable in predicting of ischemic stroke remains unclear. No specialized scoring system has so far been developed to assess 28-day mortality risk in ischemic stroke papulation. The constructed nomogram established herein was superior in predicting short-term, namely 28-day, mortality than the aforementioned clinically commonly used scoring systems. However, we did not further verify the Acute Physiology and Chronic Health Evaluation (APACHE) II and APACHE III scores in this study, owing to a limitation of the SQL language of MIMIC database, which necessitates further research exploration. The APS III score, a physiology sub-score of APACHE III, published by Knaus et al. in 1991 [26], comprises 14 indicators. Based on APS III scoring system, The C-index of model for prognosticating 28-day mortality within the ischemic stroke cohort stood at 0.761, second only to our nomogram. SAPS II, an evolution of the original SAPS by Le Gall et al. [27], represents a seminal advancement in critical care prognostication, with subsequent iterations culminating in SAPS III. However, empirical investigations have revealed a propensity for the SAPS III scoring schema to overestimate mortality rates among ICU patients afflicted with internal disorders when juxtaposed against its predecessor, SAPS II [28]. Of particular significance is the discernible superiority of our nomogram in predicting 28-day mortality within the ischemic stroke cohort when compared against SAPS II, an instrument encompassing a comprehensive array of 15 variables. This nuanced delineation underscores the unparalleled accuracy and clinical relevance engendered by our predictive model within this specific clinical context. Studies have shown that complex scoring systems require many indicators, making their application in the clinical setting difficult [29]. Six sub-score ranged from 0–5 for LODS, developed by Le Gall et al. in 1996 [30]. Previous prediction of outcomes in neurological ICU patients have shown that LODS has more stability than APACHE II [31]. However, we found that our nomogram was more superior to the LODS score, which accounts for the largest proportion of the nervous system. To date, only a handful of studies have described application of OASIS, which was developed using machine-learning algorithms with appropriate minimal number of factors [32], among neurological ICU population. Zhu et al. [25] suggested that OASIS had the highest performance in predicting mortality among neurological critically ill population. However, the present study demonstrated that OASIS, just like LODS, was less superior in predicting ischemic stroke. Based on six different scores, an agreement was reached in 1994 to create the SOFA score [33]. Rather than predicting outcomes, SOFA was intended to describe progression of critical illness complications. Currently, the system is mainly used to assess sepsis patients, with very little application in prognosis of ischemic stroke. The SOFA score was utilized to accurately predict prognosis of severe acute ischemic stroke [10]. On the other hand, the GCS score is a clinically common, easy, and non-invasive tool for assessing the degree of coma, including eye opening response, language response and body movement. As part of mortality in ischemic stroke score (MIS), lower GCS score is a predictive indicator for hospital mortality [34, 35]. Results of C-index, NRI, IDI and DCA, revealed that there was no excellent ability of SOFA and GCS to predict death among ischemic stroke.

Numerous risk factors have been associated with stroke. In the present study, 14 independent factors, namely age, ethnicity type, marital status, underlying metastatic solid tumor, CCI, heart rate, GCS, WBC, glucose concentrations, sodium concentrations, potassium concentrations, MV, use of heparin and mannitol injection, were significantly correlated with stroke mortality.

In comparison to other scoring systems clinically used under common conditions, our model demonstrates several notable differences in variable selection. For example, while GCS primarily focuses on neurological status, our model incorporates a broader range of clinical and demographic variables, including comorbidities, laboratory parameters, and treatment modalities. This broader scope allows for a more comprehensive assessment of patient outcomes by capturing the multifaceted nature of ischemic stroke. Furthermore, our model includes variables such as ethnicity type, marital status, and underlying metastatic solid tumor, which are not commonly included in existing scoring systems. These variables reflect important social and demographic determinants of health that may influence outcomes beyond traditional clinical factors. By incorporating these variables, our model aims to provide a more holistic understanding of patient mortality risk, accounting for both clinical and non-clinical factors. Additionally, our model utilizes advanced statistical techniques such as LASSO regression and 10-fold cross-validation to identify and validate the most relevant predictors of mortality. This rigorous approach ensures the robustness and generalizability of our model across different patient populations. Overall, the differences in variable selection between our model and other scoring systems highlight the unique contributions of our approach to mortality prediction in ischemic stroke. By integrating a diverse array of clinical and demographic factors, our model offers a more comprehensive and nuanced assessment of patient outcomes, with the potential to inform personalized treatment strategies and improve clinical decision-making.

In general, stroke is a disease associated with aging [15]. The brain reserve becomes weaker during the aging process, a phenomenon that worsens prognosis of older patients [36]. On the other hand, younger ischemic stroke patients may experience better outcomes [6]. Although the main effect of stroke on the brain is well recognized, infections remain the most common cause of stroke-related deaths. Impairment of intestinal barrier function, during advanced age, mediates transportation of gut-derived bacteria which predisposes the body to infections [37]. Moreover, the aging process is associated with downregulation of basal Bcl-2 expression, while neuronal injury is elevated through cell apoptosis. Therefore, aging aggravates stroke-related brain damage [38].

The impact of Hispanic ethnicity on the prognosis of ischaemic stroke remains a topic of debate [39, 40]. In our study, we developed a nomogram that revealed a lower risk of death for Hispanics compared to White, Black, Asian, and other racial groups. However, it is important to note that Hispanics face significant challenges in accessing appropriate health care and follow-up due to socioeconomic status and limited health insurance coverage [41]. While our research team consisted of only a small number of Hispanic individuals, we believe that it is essential to expand discussions of the prognosis of ischemic stroke to encompass the larger Hispanic population. In our investigation, it was discerned that individuals who were widowed exhibited inferior prognostic outcomes subsequent to an ischemic stroke event, in stark contrast to their married counterparts who demonstrated a notably more favorable trajectory, consistent with findings from our previous research [8]. Matrimony can serve as a fundamental source of social support, which can lower the likelihood of engaging in unfavorable behaviors [42] and confer stable behavioral and psychosocial resources that aid in illness prevention and treatment [43]. In contrast, losing a spouse is one of the most stressful life events one can experience, and can result in acute stress from bereavement, as well as chronic stress stemming from reduced emotional, financial, and social support [44]. The stress associated with widowhood can have deleterious effects on mental, physical, and cognitive health, as well as health behaviors, via both psychosocial and physiological pathways [44]. These findings underscore the importance of recognizing the significant impact of social support on health outcomes, and highlight the need for interventions that can mitigate the negative effects of widowhood-related stress on post-stroke recovery. By doing so, we can promote better health outcomes and improved quality of life for those who have experienced the loss of a spouse. Our regression analysis findings underscore the significance of factors like race and marital status as risk factors for poor prognosis among ischemic stroke. Their incorporation enriches the predictive capacity of our model by encompassing the multifaceted determinants of patient outcomes. It is imperative to acknowledge that excluding such factors may indeed impact the performance of the model. Therefore, we contend that the inclusion of race and marital status contributes valuable insights to the predictive model’s robustness. Moving forward, we recognize the necessity for further investigation into the specific contributions of these variables and their implications for clinical decision-making. Subsequent studies can delve deeper into elucidating the intricate interplay between clinical and non-clinical factors to enhance the predictive accuracy and applicability of our model.

Underlying metastatic solid tumor increase mortality risk in stroke population. Our results were consistent with findings from a published study, which demonstrated that cancer was one of mortality predictors in ischemic stroke [7]. In addition, tumors frequently develop at a site of chronic inflammation, while inflammation not only promotes tumor growth but also exacerbates the risk of thrombosis [45]. A previous study found that prognostic factors were associated with increased thrombin–antithrombin complex and D-dimer levels in tumor-related ischemic stroke [46]. Furthermore, as one of cAMP-response-element binding protein (CREB) transcription factor, CREB1 is downregulated in the cancer stroke group compared with the stroke group alone, making plasticity and recovery of cortical circuits difficult [47]. In this study, the CCI was utilized to evaluate comorbidity, as it is a validated tool that accounts for multiple comorbidities. Our findings demonstrated a positive correlation between CCI and mortality rates in individuals afflicted with ischaemic stroke, which is in accordance with current research, thus substantiating the significant predictive value of CCI for clinical outcomes in ischemic stroke [48]. Our investigation revealed a notable correlation between elevated maximum heart rate within the initial day following admission to the ICU and heightened susceptibility to short-term mortality among individuals afflicted with ischemic stroke. Notably, few studies have examined the impact of heart rate on post-stroke outcomes, despite its potential relevance in predicting major clinical events such as death. Specifically, heart rate parameters, including mean, maximum, and minimum values, have been identified as predictors of adverse outcomes following acute ischaemic stroke, with maximum heart rate demonstrating the strongest predictive power [49].

Patient prognoses are significantly influenced by several laboratory indicators, such as glucose, WBC, sodium concentrations and potassium concentrations. For instance, presence of high blood glucose was associated with adverse prognosis among individuals with acute ischemic stroke [50]. Higher blood glucose, as one of secondary systemic insults, can extend the zone of ischemia, a phenomenon that necessitates either prevention or correction to lower the risk of infarction progression [51]. For acute ischemic stroke, it has been shown that WBC count is a predictor of mortality at 1 month [52]. To prevent neurologic deterioration, there is need to prevent hyperglycemia [51, 53]. Interestingly, acute ischemic stroke individuals with higher blood glucose and WBC counts exhibited adverse outcomes, while prediction of hospital mortality in this population was improved by combining WBC count with blood glucose [9]. Electrolyte imbalances are frequently encountered in clinical practice, particularly among hospitalized patients, and have been linked to adverse outcomes [54]. Our study found that elevated levels of serum potassium and sodium were associated with poor prognosis among ischemic stroke. Serum sodium alterations are commonly observed in clinical practice and are often indicative of poor prognosis in hospitalized patients. Specifically, in the acute phase of ischemic stroke, higher serum sodium levels have been associated with increased risk of mortality and neurological impairment [54]. On the other hand, the relationship between hyperkalemia and short-term mortality from ischemic stroke has not been extensively explored in the literature. As a critical clinical blood biomarker, serum potassium plays a crucial role in maintaining basic cellular function. However, few studies have investigated the association between hyperkalemia and short-term mortality in ischemic stroke. Our findings highlight the importance of monitoring electrolyte imbalances, particularly serum potassium and sodium levels, in patients with ischemic stroke, as they may serve as valuable prognostic indicators.

In the present study, various interventions, such as MV, use of heparin and osmotic agents, were significant independent prognostic indices for predicting mortality, in line with previous studies [10]. MV, one of the supportive treatments for organs, plays an important effect on admission of ischemic stroke patients to the ICU [55]. In addition, MV was applied in up to 7.9% of population with ischemic stroke [56]. The need for MV is strongly correlated with mortality, and patients with ischemic stroke hospitalized on MV had a 46.8% in-hospital mortality rate [56]. A multi-center cohort study has been used to evaluate 1-year outcomes and confirm individuals with ischemic stroke who may profit by long-term MV (NCT03335995) [57]. Studies have also shown that cerebral oedema can cause neurological deterioration [51]. In fact, mannitol is commonly used to treat cerebral edema in the aftermath of stroke occurrence. As patients with ischemic stroke are prescribed hyperosmotic drugs, cerebral edema may be present and more severe. Moreover, certain types of cerebral edema are unresponsive to hyperosmotic drugs, such as cytotoxic cerebral oedema [58]. This study revealed that the application of heparin was deemed as an independent danger element of transitory mortality in individuals afflicted with ischaemic stroke. While early heparin therapy could impede the growth of ischaemic lesions in select patients with acute ischaemic stroke [59], contemporary evidence obtained from randomized trials illustrates a clinically and statistically noteworthy peril of major intracranial and extracranial haemorrhage with premature utilization of anticoagulants in people with acute ischaemic stroke [60]. Consequently, the customary utilization of any presently accessible anticoagulant for acute ischaemic stroke lacks support [60].

This study had several limitations. Firstly, the study utilized data from the MIMIC-IV database, thus there is possibility of both data bias and entry errors. Consequently, we have carefully evaluated outliers and appropriately managed missing values. Secondly, the limitation of concerning MIMIC database meant that several important variables were not included in this study. These included neuroimaging examination, electrophysiological examination, endovascular thrombectomy, NIHHS, APACHE II and APACHE III scores, among others. It is possible that unidentified confounding factors may have influenced results of the established nomogram. Thirdly, we only conducted internal validation. Further high-quality researches and studies, using external validation and test sets, are needed to validate the established nomogram.

## Conclusions

In summary, our results indicated that age, ethnicity, marital status, underlying metastatic solid tumor, CCI, heart rate, GCS, glucose, WBC, sodium concentrations, potassium concentrations, MV, application of heparin and mannitol injection are significantly associated with 28-day mortality among ischemic stroke individuals. We employed LASSO and multiple logistic regression algorithms to establish and validate a nomogram that can accurately predict short-term (28-day) mortality in ischemic stroke patients. This nomogram exhibited satisfactory discrimination, calibration and net clinical benefits, thus has potential in evaluating patient mortality. It may also help to facilitate communication of medical conditions in clinical practice. Using external validation and test sets, further studies are expected to validate the performance of the established nomogram.

## Supporting information

S1 Fig Schematic representation illustrating the patient selection process. (TIF)

S1 Table Baseline clinical features of individuals afflicted with ischemic stroke. (DOC)

## Abbreviations

ICU: intensive care unit
IQR: interquartile range
BMI: body mass index
GCS: Glasgow coma score
APS III: acute physiology score III
SOFA: sequential organ failure assessment
LODS: logistic organ dysfunction system
SAPS II: simplified acute physiology score II
OASIS: oxford acute severity of illness score
APACHE: acute physiology and chronic health evaluation
MAP: mean arterial pressure
LOS: length of stay
OR: odd ratio
CI: confidence interval
C-index: concordance index
NRI: net reclassification index
IDI: integrated discrimination improvement
DCA: decision curves analysis
LASSO: least absolute shrinkage and selection operator
MIMIC-IV: medical information mart for intensive care IV
WBC: white blood cells
AG: anion gap
BUN: blood urea nitrogen
MV: mechanical ventilation
NIHSS: national institute of health stroke scale
ICD: international classification of disease
CCI: Charlson comorbidity index
CREB: cAMP-response-element binding protein
