# Supplementary figures and images for: Development and validation of a nomogram for predicting 28-day mortality in patients with ischemic stroke
Source: PLoS One. 2024 Apr 24;19(4):e0302227. doi: 10.1371/journal.pone.0302227 (PMC11042708; doi:10.1371/journal.pone.0302227)

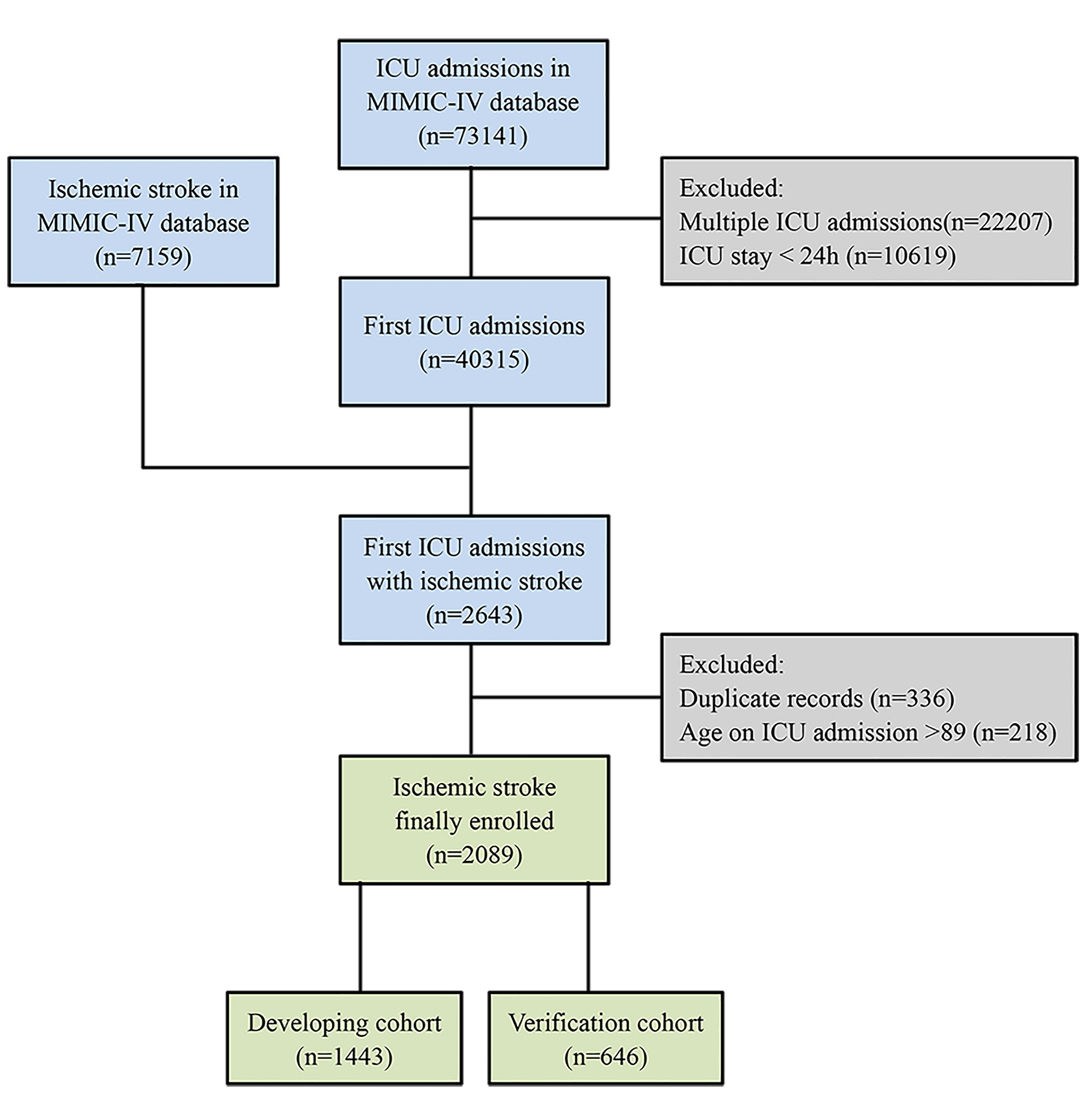

Supplement: S1 Fig — (TIF) [file pone.0302227.s001.tif]
