# Supplementary material for: Development and validation of a nomogram for predicting 28-day mortality in patients with ischemic stroke
Source: PLoS One. 2024 Apr 24;19(4):e0302227. doi: 10.1371/journal.pone.0302227 (PMC11042708; doi:10.1371/journal.pone.0302227)
Supplement: S1 Table — (DOC) [file pone.0302227.s002.doc]

**S2 Table**. Baseline Clinical Features of Individuals Afflicted with Ischemic Stroke.

| Characteristics | All patients (N=2089) | Developing cohort (n=1443) | Verification cohort (n=646) | P-value |
| --- | --- | --- | --- | --- |
| Age, median (IQR) | 68.92(57.33, 78.86) | 69.05(57.88, 78.94) | 68.42(56.50, 78.57) | 0.515 |
| Female, No. (%) | 993(47.53) | 682(47.26) | 311(48.14) | 0.710 |
| Weight, median (IQR) (kg) | 78.10(66.50, 93.50) | 78.00(66.90, 93.20) | 79.20(65.90, 93.90) | 0.982 |
| **Race,** **No. (%)** | | | | |
| White | 1242(59.45) | 854(59.18) | 388(60.06) | 0.119 |
| Hispanic | 74(3.54) | 49(3.40) | 25(3.87) |  |
| Black | 223(10.67) | 160(11.09) | 63(9.75) |  |
| ASIAN | 60(2.87) | 33(2.29) | 27(4.18) |  |
| Other | 490(23.46) | 347(24.05) | 143(22.14) |  |
| **Marital Status, No. (%)** | | | | |
| Married | 894(42.80) | 604(41.86) | 290(44.89) | 0.299 |
| Single | 516(24.70) | 356(24.67) | 160(24.77) |  |
| Widowed | 217(10.39) | 163(11.30) | 54(8.36) |  |
| Divorced | 146(6.99) | 99(6.86) | 47(7.28) |  |
| Other | 316(15.13) | 221(15.32) | 95(14.71) |  |
| **First Care Unit,** **No. (%)** | | | | |
| Medical ICU | 190(9.10) | 132(9.15) | 58(8.98) | 0.886 |
| Surgical ICU | 663(31.74) | 456(31.60) | 207(32.04) |  |
| Medical ICU/Surgical ICU | 101(4.83) | 75(5.20) | 26(4.02) |  |
| Neuro Surgical ICU | 284(13.60) | 192(13.31) | 92(14.24) |  |
| Trauma Surgical ICU | 223(10.67) | 152(10.53) | 71(10.99) |  |
| Other ICU | 628(30.06) | 436(30.21) | 192(29.72) |  |
| **Underlying Diseases,** **No. (%)** | | | | |
| Myocardial Infarct | 327(15.65) | 222(15.38) | 105(16.25) | 0.613 |
| Congestive Heart Failure | 467(22.36) | 327(22.66) | 140(21.67) | 0.616 |
| Peripheral Vascular Disease | 275(13.16) | 193(13.37) | 82(12.69) | 0.670 |
| Dementia | 69(3.30) | 53(3.67) | 16(2.48) | 0.157 |
| Chronic Pulmonary Disease | 377(18.05) | 249(17.26) | 128(19.81) | 0.160 |
| Rheumatic Disease | 57(2.73) | 40(2.77) | 17(2.63) | 0.856 |
| Peptic Ulcer Disease | 33(1.58) | 22(1.52) | 11(1.70) | 0.763 |
| Mild Liver Disease | 111(5.31) | 72(4.99) | 39(6.04) | 0.324 |
| Severe Liver Disease | 35(1.68) | 26(1.80) | 9(1.39) | 0.501 |
| Renal Disease | 321(15.37) | 220(15.25) | 101(15.63) | 0.820 |
| Diabetes without  chronic complication | 573(27.43) | 389(26.96) | 184(28.48) | 0.470 |
| Diabetes with  chronic complication | 175(8.38) | 131(9.08) | 44(6.81) | 0.084 |
| Paraplegia | 991(47.44) | 696(48.23) | 295(45.67) | 0.277 |
| Malignant Cancer | 181(8.66) | 125(8.66) | 56(8.67) | 0.996 |
| Metastatic Solid Tumor | 74(3.54) | 52(3.60) | 22(3.41) | 0.821 |
| Acquired Immune  Deficiency Syndrome | 5(0.24) | 2(0.14) | 3(0.46) | 0.159 |
| **Charlson Comorbidity Index, median (IQR)** | 7.00(5.00, 8.00) | 7.00 (5.00, 8.00) | 7.00 (5.00, 9.00) | 0.453 |
| **Disease severity Scoring System, median (IQR)** | | | | |
| Firstday GCS* | 12.00(8.00, 14.00) | 12.00(8.00, 14.00) | 12.00(8.00, 14.00) | 0.898 |
| Firstday SOFA | 4.00(2.00, 6.00) | 4.00(2.00, 7.00) | 4.00(2.00, 6.00) | 0.867 |
| Firstday LODS | 4.00(2.00, 7.00) | 4.00(2.00, 7.00) | 4.00(2.00, 7.00) | 0.831 |
| Firstday OASIS | 33.00(26.00, 40.00) | 33.00(26.00, 40.00) | 33.00(26.00, 40.00) | 0.573 |
| Firstday APS III | 42.00(30.00, 61.00) | 42.00(30.00, 61.00) | 43.00(30.00, 62.00) | 0.901 |
| Firstday SAPS II | 32.00(25.00, 41.00) | 32.00(25.00, 41.00) | 32.00(25.00, 42.00) | 0.571 |
| **Vital Indicators, median (IQR)** | | | | |
| Temperature (°C) ** | 37.28(37.06, 37.83) | 37.28(37.06, 37.80) | 37.33(37.00, 37.90) | 0.482 |
| Heart Rate (beats/min) ** | 99.00(86.00, 11300) | 99.00(87.00, 11400) | 98.00(85.00, 11200) | 0.213 |
| Respiratory Rate (breaths/min) *** | 18.65(16.84, 21.05) | 18.64(16.80, 21.10) | 18.74(17.00, 20.94) | 0.698 |
| SBP (mmHg) ** | 161.00(145.00, 178.00) | 160.00(144.00, 178.00) | 162.00(147.00, 179.00) | 0.171 |
| Oxygen Saturation (%) * | 93.00(91.00, 95.00) | 93.00(91.00, 95.00) | 93.00(91.00, 95.00) | 0.937 |
| Glucose (mmol/L) * | 5.83(4.94, 7.06) | 5.83(4.89, 7.11) | 5.78(4.94, 7.00) | 0.765 |
| Firstday Urine Output (L) | 1.60 (1.04, 2.35) | 1.61 (1.04, 2.37) | 1.58 (1.03, 2.32) | 0.547 |
| **Laboratory Indicators, median (IQR)** | | | | |
| White Blood Cells (K/uL) ** | 11.90(8.80, 15.90) | 11.90(8.70, 16.00) | 11.85(9.00, 15.60) | 0.947 |
| Hemoglobin (g/dL) * | 11.60(9.70, 13.10) | 11.50(9.60, 13.10) | 11.80(9.80, 13.20) | 0.233 |
| Hematocrit (%) * | 34.90(29.50, 39.10) | 34.70(29.40, 39.00) | 35.20(29.70, 39.40) | 0.332 |
| Platelets (K/uL) * | 195.00(149.00, 252.00) | 196.00(149.00, 254.00) | 194.00(149.00, 245.00) | 0.362 |
| BUN (mg/dL) ** | 6.78(5.00, 9.64) | 6.43(4.64, 9.64) | 6.78(5.00, 9.28) | 0.487 |
| Creatinine (μmmol/L) ** | 88.40(70.72, 114.92) | 88.40(70.72, 114.92) | 88.40(70.72, 114.92) | 0.082 |
| Sodium (mEq/L) ** | 140.00(138.00, 143.00) | 140.70(138.00, 143.00) | 140.00(138.00, 143.00) | 0.524 |
| Potassium (mEq/L) ** | 4.30(3.90, 4.70) | 4.30(3.90, 4.70) | 4.20(3.90, 4.70) | 0.346 |
| Calcium (mEq/L) * | 2.13(1.98, 2.23) | 2.13(1.98, 2.23) | 2.13(1.98, 2.25) | 0.669 |
| Chloride (mEq/L) ** | 106.00(103.00, 109.00) | 106.00(102.00, 109.00) | 106.00(103.00, 109.00) | 0.395 |
| Prothrombin Time (sec) ** | 13.10(11.90, 15.30) | 13.20(11.90, 15.50) | 13.00(11.90, 14.90) | 0.134 |
| PTT (sec) ** | 30.40(26.80, 40.60) | 30.50(26.80, 40.70) | 30.35(26.90, 40.60) | 0.871 |
| INR ** | 1.20(1.10, 1.40) | 1.20(1.10, 1.40) | 1.20(1.10, 1.38) | 0.126 |
| Anion Gap (mmol/L) * | 13.00(12.00, 15.00) | 13.00(12.00, 15.00) | 13.00(11.00, 15.00) | 0.136 |
| Bicarbonate (mmol/L) * | 22.00(20.00, 24.00) | 22.00(20.00, 24.00) | 22.00(19.42, 24.00) | 0.127 |
| **Medications and Interventions,** **No. (%)** | | | | |
| Endovascular Obstruction Removal | 192(9.19) | 131(9.08) | 61(9.44) | 0.790 |
| Alteplase | 38(1.82) | 24(1.66) | 14(2.17) | 0.426 |
| Antiplatelet | 559(26.76) | 394(27.30) | 165(25.54) | 0.400 |
| Anticoagulation | 688(32.93) | 477(33.06) | 211(32.66) | 0.860 |
| Furosemide | 93(4.45) | 68(4.71) | 25(3.87) | 0.388 |
| Mannitol | 83(3.97) | 55(3.81) | 28(4.33) | 0.572 |
| Vasoactive Agent | 515(24.65) | 355(24.53) | 161(24.92) | 0.848 |
| Invasive Mechanical Ventilation | 756(36.19) | 524(36.31) | 232(35.91) | 0.860 |
| Supplemental Oxygen | 794(38.01) | 552(38.25) | 242(37.46) | 0.730 |
| Renal Replacement Therapy | 55(2.63) | 39(2.70) | 16(2.48) | 0.766 |
| Intracranial Pressure Monitor | 85(4.07) | 57(3.95) | 28(4.33) | 0.681 |
| **Outcomes** |  |  |  |  |
| 28-Day Mortality (%) | 457(21.88) | 311(21.55) | 146(22.60) | 0.592 |
| ICU Mortality (%) | 269(12.88) | 172(11.92) | 97(15.02) | 0.051 |
| Hospital Mortality (%) | 378(18.09) | 246(17.05) | 132(20.43) | 0.063 |
| ICU LOS (days) | 3.81(1.98, 7.78) | 3.85(2.00, 7.89) | 3.54(1.96, 7.52) | 0.589 |
| Hospital LOS (days) | 9.04(5.03, 17.54) | 9.18(5.03, 17.27) | 8.95(5.03, 17.70) | 0.558 |

ICU, Intensive Care Unit; IQR, Interquartile Range; GCS, Glasgow Coma Scale; APS III, Acute Physiology Score III; SOFA, Sequential Organ Failure Assessment; LODS, Logistic Organ Dysfunction System; SAPS II, Simplified Acute Physiology Score II; OASIS, Oxford Acute Severity of Illness Score; SBP, Systolic Blood Pressure;BUN, Blood Urea Nitrogen; PTT, Partial Thromboplastin Time; INR, International Normalized Ratio; LOS, Length of Stay; *: the min value of indicators on the firstday of ICU stay; **: the max value of indicators on the firstday of ICU stay; ***: the mean value of indicators on the firstday of ICU stay. Antiplatelet was defined as the use of aspirin, clopidogrel or dipyridamole within 24 hours after ICU admission. Anticoagulation was defined as the use of heparin, warfarin, rivaroxaban, argatroban, apixaban, bivalirudin, or dabigatran within 24 hours after ICU admission. Vasoactive Agent was defined as the use of norepinephrine, epinephrine, phenylephrine, dopamine, dobutamine, vasopressin or milrinone within 24 hours after ICU admission. Disease severity scoring system, vital indicators, laboratory indicators and interventions were evaluated within 24 hours after ICU admission.
